# Biometric Strategies to Improve Vaccine Immunogenicity and Effectiveness

**DOI:** 10.3390/biomimetics10070439

**Published:** 2025-07-03

**Authors:** Vicente Javier Clemente-Suárez, Laura Redondo-Flórez, Alvaro Bustamante-Sánchez, Alexandra Martín-Rodríguez, Rodrigo Yáñez-Sepúlveda, Jose Francisco Tornero-Aguilera

**Affiliations:** 1 Faculty of Medicine, Health and Sports, Universidad Europea de Madrid, 28670 Villaviciosa de Odón, Spain; vctxente@yahoo.es (V.J.C.-S.); alvaro.bustamante@universidadeuropea.es (A.B.-S.); 2 Grupo de Investigación en Cultura, Educación y Sociedad, Universidad de la Costa, Barranquilla 080002, Colombia; 3 Department of Health Sciences, Faculty of Biomedical and Health Sciences, Universidad Europea de Madrid, 28670 Madrid, Spain; lauraredondo_1@hotmail.com; 4 Faculty of Education Sciences, UNIE University, 28015 Madrid, Spain; 5 Faculty of Education and Social Sciences, Universidad Andres Bello, Viña del Mar 2520000, Chile; rodrigo.yanez.s@unab.cl; 6 Graduate School of Business, ESAN University, Alonso de Molina 1652, Santiago de Surco, Lima 15023, Peru; doctorneroaguilera@gmail.com

**Keywords:** biomimetics, vaccine immunogenicity, bioinspired delivery systems, biomimetic adjuvants, host-microbiota interactions, evolutionary medicine

## Abstract

**Background:** Vaccines have revolutionized disease prevention, yet their effectiveness is challenged by variable immunogenicity, individual response differences, and emerging variants. Biomimetic strategies, inspired by natural immune processes, offer new avenues to enhance vaccine performance. **Objectives:** This narrative review examines how bioinspired approaches—grounded in evolutionary medicine, immunology, and host–microbiota interactions—can improve vaccine immunogenicity and long-term protection. We further examine the evolutionary foundations of immune responses, highlighting how an evolutionary perspective can inform the development of durable, broadly protective, and personalized vaccines. Furthermore, mechanistic insights at the molecular and cellular level are explored, including Toll-like receptor (TLR) engagement, dendritic cell activation pathways, and MHC-I/MHC-II-mediated antigen presentation. These mechanisms are often mimicked in biomimetic systems to enhance uptake, processing, and adaptive immune activation. **Results:** The review highlights how immunosenescence, maternal immunity, genetic variation, and gut microbiota composition influence vaccine responses. Biomimetic platforms—such as nanoparticle carriers and novel adjuvants—enhance antigen presentation, boost adaptive immunity, and may overcome limitations in traditional vaccine approaches. Additionally, co-administration strategies, delivery systems, and microbiota-derived immunomodulators show promise in improving vaccine responsiveness. **Conclusions:** Integrating biomimetic and evolutionary principles into vaccine design represents a promising path toward safer, longer-lasting, and more effective immunizations

## 1. Introduction

Vaccines have revolutionized modern medicine by effectively preventing infectious diseases and safeguarding public health. In light of global health disparities and emerging infectious threats, the review of biomimetic and physiological strategies for vaccine enhancement provides essential guidance for both research innovation and public policy. These strategies are not only promising at the experimental level but also hold the potential to be integrated into immunization campaigns worldwide, particularly in low- and middle-income countries (LMICs) where vaccine responsiveness remains a challenge [1].

In this comprehensive narrative review, we aim to explore how biomimetic principles can unravel the intricacies surrounding vaccine immunogenicity and effectiveness, identifying nature-inspired strategies to enhance immune responses and develop more sustainable vaccination approaches. We will explore various aspects, ranging from the immune response mechanisms triggered by vaccines to the duration of vaccine-induced immunity [2]. Additionally, we will delve into the variability observed in vaccine responses among individuals and its impact on overall vaccine effectiveness [3]. The role of vaccination in mitigating disease transmission will be examined, highlighting the importance of achieving herd immunity and the challenges posed by emerging variants of concern [4]. Furthermore, we will explore the influence of immunosenescence on vaccine responses in older adults and the significance of maternal vaccination in conferring neonatal immunity [5].

Nonetheless, limitations such as sample heterogeneity, regional disparities in microbiome composition, and the generalizability of immunogenicity findings should be recognized to maintain a balanced interpretation of the literature [6,7]. Additionally, biomimetic vaccine platforms may pose significant challenges, including complex manufacturing processes, stability issues, and scalability concerns. The potential for unintended immune responses or reduced safety profiles must also be critically evaluated before widespread application.

To conduct this narrative review, we conducted a systematic literature review to gather the available literature on unraveling the complexities of vaccine immunogenicity and effectiveness. Various search methods and strategies were employed to identify relevant studies. For the literature search, we utilized both primary sources, such as scientific articles, and secondary sources, including bibliographic indexes, web pages, and databases. PubMed, SciELO, and Google Scholar were the main search engines used. We focused on articles published from 1 January 2013. Additionally, we examined the reference lists of all identified studies obtained through the methods. Studies that utilized outdated data, covered irrelevant topics, or did not align with the specific purpose of our study were excluded following previous narrative reviews [8,9,10,11,12].

To ensure studies met the inclusion criteria, the review authors independently screened the titles and abstracts of all retrieved records. The selection of studies was conducted in a collaborative manner, and the results were thoroughly discussed to shape the narrative review. The extraction of information was carried out by the same review authors involved in the study selection process.

## 2. Immunogenicity and Vaccine Response

These studies enable scientists to assess the suitability of a vaccine against various viral strains and determine the correct vaccine dosage. A key implication derived from these studies is the need for booster doses, which are essential to maintaining long-term immunity, especially in the face of waning responses and emerging viral mutations [13].

Active immunization involves administering all or part of a microorganism or a modified product derived from it, such as a toxoid, purified antigen, or genetically engineered antigen. This process triggers an immune response that emulates the natural infection but poses minimal or no risk to the beneficiary [14]. Immunization induces various forms of protective responses, including the production of antitoxins, antibodies that inhibit adherence or invasion, neutralizing antibodies, as well as other forms of humoral or cellular immune responses in the recipient. The level of protection provided by immunization varies, with some immunizing agents offering complete, nearly lifelong protection against the disease, others providing partial protection, and some requiring repeated administration at regular intervals [15].

### 2.1. Types of Vaccines

A vaccine is a biological product that can be used to induce an immune response that, upon subsequent exposure to a pathogen, confers protection against infection and/or disease [16]. This can be achieved if the vaccination contains antigens that are either directly derived from the disease or are synthetically generated to represent components of the pathogen. One or more protein antigens that elicit protective immune responses are crucial components of most vaccinations. Polysaccharide antigens, on the other hand, can provoke protective immune responses and have been utilized as the foundation for vaccines created since the late 1980s to ward off a variety of bacterial illnesses, such as Streptococcus pneumoniae pneumonia and meningitis [17].

**Figure 1:**
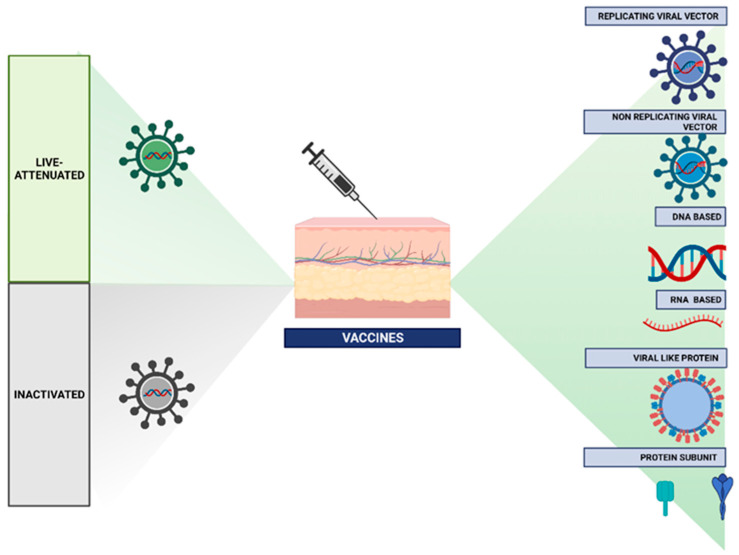
Different varieties of vaccines based on their composition, beginning with the distinction between live and attenuated organisms, as well as viral vector or DNA and RNA bases.

To differentiate vaccines containing attenuated replicating strains of the relevant pathogenic organism from vaccines containing only pathogen components or destroyed complete organisms, vaccines are often categorized as live or non-live (also referred to loosely as ‘inactivated’). Several platforms have been created during the past few decades in addition to ‘conventional’ live and non-live vaccinations. These include non-replicating or replicating viral vectors, nucleic acid-based RNA and DNA vaccines, and virus-like particles or protein subunits [18] (Figure 1).

#### Vaccines, Action, and Reaction

Therefore, as a virus infects the body, it will be detected by the immune system, the body’s ‘detect and destroy’ defense force. Immune systems have evolved to detect previously unknown pathogens (such as viruses) [19]. As mentioned, live or inactivated microorganisms may be included in vaccines derived from an intact infectious agent [17]. Active infection results from viral replication following administration, but the host experiences minimal or no adverse effects. Vaccines against certain viruses and virtually all bacteria are inactivated (killed) preparations made of subunits (purified components), or by chemical means are deconjugated with immunobiologically active proteins (such as tetanus toxoids) [20]. In subunit, inactivated, and conjugated formulations, viruses and bacteria are incapable of multiplication. Protein-based vaccines frequently comprise proteins from the surface of a virus. These proteins enable the virus to attach to human cells and infect them [20]. In a vaccine, however, the laboratory-produced proteins only stimulate the immune system and do not cause infection or disease. Likewise, mRNA and viral-vector vaccines contain instructions for human cells on how to produce an antigen protein in lieu of a protein. These directions are available in one of two formats. Firstly, a molecule known as messenger ribonucleic acid, or mRNA [21]. Secondly, genetic information is contained within a harmless ‘vector’ or carrier virus that has been altered so that it cannot cause disease. When a person receives mRNA or a vaccine containing a viral vector, some of their cells interpret these instructions [21]. These cells then produce the antigen protein for a brief time before degrading the mRNA or the benign virus. The immune system recognizes antigens produced by the body’s own cells as foreign, activating immune cells and producing antibodies [22,23,24].

Even though the development of effective vaccines has saved innumerable lives from infectious diseases, the complexity of the human immune system necessitated the creation of animal models, such as inbred mice, to define the mechanisms of immunity [25]. Recently, new strategies and technologies have been devised to investigate the human immune system directly with unprecedented precision [25,26]. Vaccines and autoimmunity are closely interconnected disciplines. The effectiveness of a vaccine relies on its ability to elicit a memory T-cell response in the host’s immune system over time. T cells, also known as T lymphocytes, form a significant part of the adaptive immune system, performing various functions such as directly eliminating infected host cells, activating other immune cells, producing cytokines, and regulating the immune response [26]. While vaccines can induce an immune response, it is important to note that most of the adverse effects observed so far have been transient and acute [27].

The impact of vaccination on global health cannot be overstated, as highlighted by Plotkin et al. in the 1980s and continues to hold true today [16]. Safe water is the only intervention that has had such a profound effect on population growth and mortality reduction [16]. However, providing vaccines to all children and vulnerable populations worldwide still poses challenges, especially for communities that are difficult to access due to geographical, political, or cultural barriers. Overcoming these obstacles requires sustained commitment and dedication on an international, governmental, and individual level [28]. In this context, B-cells (B lymphocytes) play a crucial role as a type of white blood cell responsible for producing antibodies, essential infection-fighting proteins. They are a vital component of the immune system, protecting the body against harmful pathogens, such as viruses, bacteria, and parasites [29]. Autoimmunity involves the breakdown of tolerogenic checkpoints, and emerging evidence suggests that the metabolic state of B cells may contribute to this dysfunction. Investigating the metabolic phenotype of B cells in autoimmunity is an active area of research, and targeting metabolic alterations may hold promise as a therapeutic approach [30].

The multifaceted role of T cells in immunity is of utmost importance for mounting effective immune responses. While their involvement in B cell development and antibody production in lymph nodes is well-established, T cells also play a central role in coordinating and regulating the immune system’s response to pathogens. Extensive research on individuals with inherited or acquired immunodeficiency has revealed the critical role of T cells in controlling infections. Studies have shown that while antibody deficiency increases susceptibility to infection, T cell deficiency presents a different challenge by impairing the ability to control a pathogen once an infection has occurred [31]. This underscores the significance of T cells in immune defense beyond their involvement in antibody production. Overall, these interconnected aspects of vaccines, autoimmunity, B cells, and T cells highlight the complexity of the immune system and the importance of continued research and dedication to improve public health on a global scale.

However, it is important to note that our understanding of T cell functions in immunity is continuously evolving. Ongoing research is uncovering new insights into the diverse roles of T cells, such as their contribution to immune memory, cytotoxic responses, and the regulation of immune-inflammatory processes. These findings deepen our understanding of the intricate interplay between T cells and other components of the immune system.

Even though it will be explained in detail in subsequent sections, the immune system (comprised of B-cells and T-cells) is typically very effective at identifying and eliminating viruses. Despite the success of vaccines over the past two centuries, people tend to focus on the potential adverse effects of immunization rather than the devastating effects of diseases that can now be avoided thanks to vaccines [32]. Autoimmunity is induced by vaccination, natural immunity is preferable to vaccine-generated immunity, and vaccines cause antigenic overload. Cytokine production, anti-idiotypic networks, expression of human histocompatibility leukocyte antigens, surface antigen modification and induction of novel antigens, molecular mimicry, bystander activation, epitope spreading, and polyclonal activation of B cells are all potential mechanisms by which vaccines could induce autoimmunity. There is convincing evidence that none of these processes play a significant role in triggering autoimmune disorders [27,33]. Thus, vaccines that stimulate both B-cells and T-cells provide a more robust immune response.

## 3. Immune Response Mechanisms

The physiological response of the body to substances it perceives as foreign or potentially dangerous. Antigens (often proteins) on the surface of substances or microbes like bacteria or viruses are recognized by the immune system, prompting an attack and destruction of, or attempt at destruction by, the immune system [34]. To maintain healthy tissue and organ function, the host must tolerate and keep in check a diverse microbial community that includes both obligatory pathogens and beneficial, commensal species. There is a wide variety of strategies employed by pathogenic microorganisms to multiply, disperse, and endanger healthy host processes [35]. Simultaneously with eliminating pathological microbes and toxic or allergenic proteins, the immune system must avoid responses that cause excessive harm to self-tissues or may eliminate beneficial, commensal microbes [36]. To control and, in most cases, eradicate these pathogens and pollutants, it comes as no surprise that the immune system employs a wide variety of defensive measures [37]. As a general characteristic of the immune system, these mechanisms depend on detecting structural characteristics of the pathogen or toxin that distinguish it from host cells. This distinction between host and pathogen or host and toxin is necessary for the host to eradicate the threat without harming its own tissues [38].

Mechanisms for identifying pathogens, toxins, or allergens fall into two broad classes. To begin, there are innate reactions programmed into the host’s DNA that may identify chemical patterns shared by many pathogens and toxins, but which are absent in the mammalian host itself. This is the immune system reacting. Second, the somatic rearrangement of gene products results in the assembly of antigen-binding molecules that are exquisitely specific for the antigens they bind to and their reactions [35]. That is the adaptive immune response. Therefore, it is crucial that the immune response be able to stop itself from using these harmful mechanisms on the mammalian host’s own tissues. In this regard, as mentioned, T cells have evolved a sophisticated mechanism that simultaneously recognizes foreign antigens and self-antigens as a molecular complex, as recognition of infected host cells by viruses, intracellular bacteria, or other intracellular parasites is a crucial function of the T cell arm of the immune system [39]. The adaptive immune system, in contrast to the innate defense mechanisms, demonstrates high levels of specificity towards its antigen targets. T- and B-lymphocyte surface antigen-specific receptors are the primary basis for adaptive immune responses [40]. Somatic rearrangement of germ-line gene elements results in the formation of complete T cell receptor (TCR) and B cell antigen receptor (Ig) genes, which encode the antigen-specific receptors of the adaptive immune response [40].

### 3.1. Immune System Cells

The creation of all immune system cells begins with a pluripotent hematopoietic stem cell, which differentiates into a common myeloid progenitor cell or a common lymphoid progenitor cell. B cells, T cells, NK cells, and NK-T cells are the four primary mature lymphocyte populations that develop from the common lymphoid progenitor [41]. In addition to several types of granulocytes, megakaryocytes, platelets, and erythrocytes can be derived from myeloid stem cells (also called common myeloid progenitors). Cells of the granulocyte lineage, including neutrophils, monocytes, macrophages, eosinophils, basophils, and mast cells, play essential roles in the immune system [42]. The production of immunologically active chemicals and the accumulation of those compounds under certain clinical situations have led to inferences about the immune roles of these granulocytes. For instance, neutrophils generate significant amounts of cytotoxic reactive oxygen species that are lethal to bacterial pathogens [43].

Additionally, some of the functions performed by these cells are, for example, that activated macrophages produce copious quantities of IFN-γ, interleukin 6 (IL-6), interleukin 12 (IL-12), and tumor necrosis factor (TNF) and exhibit potent pro-inflammatory and antibacterial activities [44]. In the presence of glucocorticoid hormones, interleukin 4 (IL-4), interleukin 10 (IL-10), or interleukin 13 (IL-13) induce alternatively activated macrophages, which exhibit anti-inflammatory functions via their own production of interleukin 10 (IL-10), the interleukin 1 (IL-1) receptor antagonist, and transforming growth factor β (TGF β) [45]. In this line, neutrophils are known to produce significant quantities of the cytokines TNF and interleukin 12 (IL-12), as well as several chemokines. This suggests that neutrophils have an additional immunoregulatory function [46].

**Figure 2:**
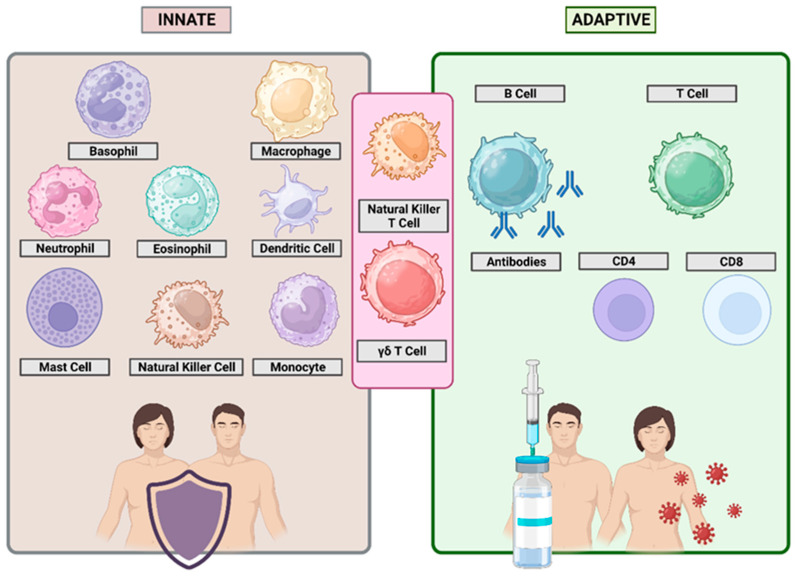
Cells of each type of immune response, innate or adaptive, and the interaction that occurs between the two systems.

Although the actions of T cells and B cells have been described previously and will be discussed in greater detail below, the immune system pursues active immunization via its actions. This refers to the production of antibodies against a specific antigen or pathogen after exposure. It can be acquired through natural infection with a microbe or vaccination (Figure 2). Active immunization is frequently ineffective without the use of “adjuvants” that enhance the immune system’s ability to respond to antigen injection [47].

### 3.2. Innate and Adaptive Immune Response

The degree to which these two branches of the immune system work together once a pathogen has invaded the host is a crucial factor in determining the host’s resistance or vulnerability. Therefore, immunologists have a lot invested in studying the cooperative interactions between innate and adaptive immune cells (Figure 2) [48]. The innate immune system works in tandem with the adaptive immune system to control infection, limit viral entrance, translation, replication, and assembly, recognize and eliminate contaminated cells, and speed up the adaptive immune response. Pathogen-associated molecular patterns (PAMPs) are recognized by pattern recognition receptors (PRRs) on the cell surface, in the endosome, and in the cytosol to initiate inflammatory responses and programmed cell death that control viral infection and facilitate clearance [49]. Thus, the innate immune system has evolved to recognize and respond to persistent pathogens based on shared genetic characteristics. Dendritic cells (DCs), macrophages, neutrophils, and other cells are all examples of the innate immune system. The T and B lymphocytes that make up the adaptive immune system, on the other hand, use antigen receptors that are not inherited but rather developed anew in everyone. As a result, the immune system’s adaptive responses are very targeted [50].

Rapid recruitment of immune cells to sites of infection and inflammation is made possible by innate immunity’s ability to synthesize cytokines and chemokines, which are tiny proteins involved in cell-cell communication and recruitment. The release of cytokines during innate immunity not only triggers local cellular responses to infection or injury but also activates many other defense mechanisms across the body [51]. The inflammatory cytokines TNF, IL-1, and IL-6 are released early in the body’s response to a bacterial infection. Concretely, Tanaka et al. specified that dysregulated, ongoing production of IL-6 has a detrimental impact on chronic inflammation and autoimmunity, even though its expression is tightly regulated by transcriptional and posttranscriptional processes [52]. Indeed, initiating cell recruitment and local inflammation, both necessary for the clearance of many infections, are functions of these cytokines. In addition, they help trigger a high body temperature [53]. These inflammatory cytokines are important therapeutic targets because their dysregulated production is typically linked to inflammatory or autoimmune illness [47]. To recognize and neutralize bacteria and other pathogens, the body employs a biochemical cascade known as the complement system. It destroys some pathogens and infected cells directly and makes them more amenable to phagocytosis, the process by which immune cells ingest germs and eliminate cell debris [54]. The innate immune system’s phagocytic function aids in the digestion of foreign substances and the disposal of dead cells or antibody complexes. Antigen-presenting cells (APCs), among them macrophages, B Cells and dendritic cells, can be mobilized and activated, triggering an adaptive immune response [55].

Otherwise, in adaptive response, T cells originate in the bone marrow’s hematopoietic stem cells and migrate to the thymus for maturation. The TCR is a family of membrane-expressed antigen-binding receptors. Different subpopulations of T cells develop, each with its own unique set of effector activities. Selective surface expression of CD4 or CD8 defines the primary subgroups. Brummelman et al. specified the differences, pointing out that CD4 lymphocytes take the lead in fighting infections. However, CD8 cells can destroy cancer cells and other invaders [56]. Each T cell has the potential to quickly divide and specialize in response to certain signals. T cells express a single kind of TCR [57]. APCs are necessary for T cells to recognize a specific antigen, as was previously indicated [58]. The major histocompatibility complex (MHC) is a set of proteins found on the surface of APCs. Class I MHC is present on all nucleated cells, while class II MHC is exclusive to macrophages, dendritic cells, and B cells in the immune system. When it comes to presenting peptides to T cells, class I MHC molecules present intracellular peptides, while class II molecules on APCs present extracellular peptides [59]. The MHC protein presents peptide antigens when a cell is infected with a virus or has phagocytized a foreign protein or organism [60]. In a recent review, Gaudino et al. specified that while great progress has been made towards understanding APC-T cell interactions, there are still various areas where research must be addressed. To begin, more research needs to be performed to understand how bacteria affect APC interactions with immune cells during the fight against microbial illnesses. Improvements in knowledge are also needed about the relationship between microbiota-derived signaling and the development of malignancies [55].

T cells possess a vast variety of TCRs, each of which is capable of binding to a different foreign peptide. T cells that are programmed to respond to harmless antigens occurring naturally in the body are essentially wiped out throughout immune system development. As soon as T cells encounter an APC that has digested an antigen and is showing the appropriate antigen fragments (peptides) linked to its MHC molecules, the T cells become activated [48,57]. T cells circulate throughout the body (through the lymphatic system and bloodstream) and accumulate (along with APCs) in lymph nodes, increasing the chances that the right T cells will come into contact with an APC carrying the appropriate peptide MHC complex. TCR activation by the MHC-antigen complex leads to the production of cytokines that further regulate the immune response [37]. T cells are largely prompted to develop into either cytotoxic T cells (CD8+ cells) or T-helper (Th) cells (CD4+ cells) by the antigen presentation pathway. CD8+ cytotoxic T cells are responsible for eliminating infected cells caused by pathogens like viruses and cancer cells displaying suitable antigens [61]. The TCR on these cells becomes active when it recognizes a peptide presented by an MHC class I molecule. Effector cells, created by the clonal proliferation of cytotoxic T cells, secrete chemicals that cause the target cells to commit apoptosis. Most effector cells perish and are removed by phagocytes when an infection clears up. Some of these cells, however, are kept as memory cells and can rapidly undergo differentiation into effector cells the next time they come into contact with the same antigen [62]. Furthermore, recent experimental models involving various organs such as the kidney, liver, gut, brain, and heart have demonstrated the importance of T cells as mediators of ischemia-reperfusion injury. These findings, as highlighted by Rabb et al., offer novel insights into the pathogenesis of ischemic acute renal failure and suggest potential therapeutic strategies. Importantly, T cells exhibit functional potential beyond adaptive immunity, as evidenced by their role as mediators of early alloantigen-independent tissue damage, thus bridging the innate immunological response with their versatile capabilities [63]. T lymphocytes traditionally belong to the adaptive immune system. T cells have been shown to be important mediators of ischemia-reperfusion injury in recent experimental models of kidney, liver, gut, brain, and heart. Rabb et al. showed that these findings provide novel insights into the pathogenesis of ischemic acute renal failure and identify novel and practicable therapeutic strategies. Moreover, T cells’ functional potential extends beyond adaptive immunity and into the realm of innate immunological response, as shown by their discovery as mediators of early alloantigen-independent tissue harm [63]. Otherwise, the findings suggest that T cells contribute to the response to immune checkpoint blockade in patients with HLA-class-I-negative DNA mismatch repair-deficient (MMR-d) colon malignancies, highlighting the potential of γδ T cells in cancer immunotherapy [64].

In this, an adaptive response, B cells, in contrast to T cells, do not require APCs to recognize antigens because of the specific antibodies produced on their cell surface. After developing from hematopoietic stem cells in the bone marrow, B cells exit the marrow with their own specialized antigen-binding receptor [29]. B cells must undergo further differentiation to fulfil their primary function of producing antibodies against foreign antigens. B cells can also serve as APCs under specific conditions. Memory B cells are “long-lived” because they retain the ability to express antigen-binding receptors after being exposed to an infection. When re-exposed to an antigen, these cells can quickly produce antibodies and clear it off. Antigen-specific receptor-activated B cells proliferate and develop into antibody-secreting plasma cells or memory B cells in response to exposure to foreign antigens. Hoffman et al. reported, for instance, that there has been a lot of progress made in targeting B cells as a means of curing kidney and other organ immunological disorders. The best therapeutic response to B-cell-targeted treatments is not yet known, however, because much is still unknown about B-cell biology [65].

The two systems are interlinked. Cooper et al. demonstrated that Natural killer (NK) cells are innate immune lymphocytes capable of killing target cells and producing immunoregulatory cytokines [66]. In this regard, new research has shown that NK cells operate outside of the typical limits of innate and adaptive immunity. It was previously believed that adaptive immunity was the only type of immunity capable of producing memory-like responses, but new research has shown that NK cells can do the same thing. NK cells have been found in several organs, and a subset of these cells, NK-22s, has been recently reported in mucosal-associated lymphoid tissue as a specialist in the synthesis of the T(H)17 cytokine IL-22 [66,67]. To fight off microbial infections, the immune system employs a wide variety of strategies. Each of these mechanisms works in tandem with the others to produce an immune response that is both broad in scope and highly targeted to the invading pathogen. Immunomodulatory therapies, vaccine development, and collateral tissue damage can all benefit from a deeper understanding of the interconnections between the various immune effector pathways [32,35,50].

## 4. Duration of Vaccine-Induced Immunity

Duration of vaccine-induced immunity is a critical consideration in the realm of infectious disease control and prevention. Vaccines play a pivotal role in stimulating the immune system to generate a protective response against specific pathogens. Understanding the longevity of this vaccine-induced immunity is essential for effective vaccine scheduling, booster dose administration, and overall population health management [68]. While the duration of vaccine-induced immunity is of utmost importance in the context of COVID-19, it is also a significant aspect for other infectious diseases. For instance, vaccines against diseases like influenza, polio, hepatitis, and human papillomavirus (HPV) are essential tools in public health programs. The ability of these vaccines to confer long-lasting protection is a key determinant in shaping vaccination strategies and reducing disease burden.

Studies on various vaccines have provided insights into the durability of immune responses. For instance, certain vaccines, like those against measles and mumps, have been shown to provide lifelong immunity in the majority of vaccinated individuals [13]. However, other vaccines necessitate periodic booster doses to maintain protective immunity. For example, tetanus and pertussis vaccines require booster shots to reinforce immunity over time [13]. To ensure optimal vaccine effectiveness, ongoing research is conducted to monitor the duration of vaccine-induced immunity for various diseases, not just COVID-19. Longitudinal studies involving vaccinated individuals are essential to assess the persistence of immune responses and evaluate any potential waning of protection over time [69]. Understanding these dynamics informs public health decisions, including the timing and necessity of booster doses.

Besides the impact of time on vaccine-induced immunity, other factors can influence the duration of protection. The characteristics of the pathogen itself and the design of the vaccine formulation play significant roles [70]. For instance, some pathogens may evolve and change over time, necessitating updated vaccines to combat new strains. As mentioned earlier, the emergence of variants such as the Alpha, Beta, Gamma, and Delta variants of the SARS-CoV-2 virus has raised concerns about their potential to evade immune responses elicited by prior infection or vaccination [71]. Continued surveillance and studies are vital to assess the effectiveness of existing vaccines against these variants and to develop updated vaccine formulations if necessary.

Furthermore, individual variation in immune response can affect the duration of vaccine-induced immunity. Factors like age, underlying health conditions, and genetic predispositions can influence the strength and persistence of immune responses. Understanding these variables helps tailor vaccination strategies to different population groups, ensuring optimal protection. The duration of vaccine-induced immunity is a multifaceted topic that extends beyond COVID-19 to encompass various infectious diseases. While some vaccines offer lifelong immunity, others necessitate booster doses. Ongoing research and surveillance play a critical role in determining the durability of vaccine-induced immunity and aid in shaping effective vaccination strategies to protect public health [72].

### 4.1. Characteristics of the Pathogen

One crucial factor is the pathogen itself. Different pathogens have varying abilities to evade or stimulate the immune system. Some pathogens, such as the viruses that cause measles and mumps, elicit a robust and long-lasting immune response, leading to lifelong immunity in most individuals who receive the vaccine [73]. These vaccines provide durable protection against subsequent infections, contributing to their effectiveness in preventing outbreaks. On the other hand, certain vaccines require periodic booster doses to maintain immunity. For example, vaccines against tetanus and pertussis (whooping cough) typically require booster shots throughout an individual’s life. The initial vaccine primes the immune system, but over time, the immune response may gradually weaken, necessitating booster doses to reinforce and refresh the immune memory [74]. This strategy ensures continued protection against these pathogens and prevents potential infections. In the case of the SARS-CoV-2 virus, which causes COVID-19, the duration of vaccine-induced immunity is still being studied. Initial evidence suggests that COVID-19 vaccines, such as those based on mRNA technology, provide strong and long-lasting protection against severe disease and hospitalization. However, the exact duration of immunity is yet to be determined, as ongoing research is evaluating the persistence of immune responses over time. This information is critical for understanding the need for booster doses or additional vaccinations in the future.

### 4.2. Formulation of the Vaccine

The formulation of a vaccine refers to the specific components and strategies used to develop the vaccine and stimulate an immune response. Different vaccine formulations employ various approaches to elicit an immune response against a particular pathogen.

One type of vaccine formulation involves using live attenuated pathogens. These vaccines are created by modifying disease-causing microorganisms to reduce their virulence while still maintaining their ability to replicate and stimulate an immune response. Live attenuated vaccines closely mimic natural infection, allowing the immune system to mount a comprehensive response. This approach can result in long-lasting immunity since it stimulates both the humoral (antibody-mediated) and cellular arms of the immune system. Examples of vaccines that utilize live attenuated pathogens include the measles, mumps, rubella (MMR) vaccine and the oral polio vaccine [75,76].

Another vaccine formulation involves using inactivated pathogens. In this approach, the disease-causing microorganisms are killed or inactivated through physical or chemical processes, rendering them unable to replicate. Inactivated vaccines typically contain whole or fragmented pathogen particles or components. While these vaccines cannot cause disease, they can still stimulate an immune response. Inactivated vaccines primarily induce a humoral immune response, stimulating the production of antibodies. Examples of inactivated vaccines include the hepatitis A vaccine and the influenza vaccine [75].

Some vaccines utilize specific protein fragments or subunits of the pathogen. These subunit vaccines contain only the components of the pathogen that are necessary to stimulate an immune response. By focusing on specific protein fragments or antigens, these vaccines can target key components of the pathogen without including unnecessary or potentially harmful components. Subunit vaccines are generally safe and can be used in individuals with weakened immune systems. Examples of subunit vaccines include the human papillomavirus (HPV) vaccine and the acellular pertussis vaccine [77].

Furthermore, advancements in vaccine technology have led to the development of nucleic acid-based vaccines. These vaccines utilize genetic material, such as DNA or RNA, that encodes specific viral or bacterial proteins. The genetic material is taken up by host cells, which then produce the pathogen’s proteins. This approach stimulates an immune response against these proteins, leading to protection against the pathogen. mRNA vaccines, such as the COVID-19 vaccines developed by Pfizer-BioNTech and Moderna, are an example of nucleic acid-based vaccines [78].

Thus, different vaccine formulations may stimulate different types of immune responses, including the production of antibodies, activation of T cells, or the induction of memory cells. The durability of the immune response can vary depending on the vaccine formulation, with some formulations providing long-lasting immunity while others may require booster doses to maintain protection. Moreover, the choice of formulation depends on various factors, including the characteristics of the pathogen, the desired immune response, safety considerations, and the effectiveness of the vaccine in inducing protection. By tailoring the formulation to the specific requirements of the target pathogen, vaccine developers can optimize the immune response and enhance the durability of vaccine-induced immunity.

### 4.3. Individual Response

The individual’s immune response to vaccination is an important factor that can influence the duration of vaccine-induced immunity. Each person’s immune system is unique and can vary in its ability to generate an effective response to vaccines.

Age is one of the factors that can affect the immune response to vaccination. In general, younger individuals tend to have more robust immune systems, which can result in a stronger and more durable response to vaccines. On the other hand, the immune response in older adults may be weaker or less persistent. This age-related decline in immune function, known as immunosenescence, can impact the duration of vaccine-induced immunity [79]. Therefore, vaccine effectiveness and duration of protection may differ between different age groups. Yet, this will be further discussed in the review.

Certain medical conditions, such as autoimmune disorders or immunodeficiency disorders, can impair the immune system’s ability to mount a robust response to vaccines. Consequently, individuals with compromised immune systems may have a reduced duration of vaccine-induced immunity compared to those with a fully functioning immune system [80].

Genetic variations among individuals can also play a role in the immune response to vaccines. The genetic makeup of an individual can influence how their immune system recognizes and responds to the vaccine components. Certain genetic variations can impact the production of antibodies, the activation of immune cells, or the generation of memory responses [81]. For example, the human leukocyte antigen (HLA) genes are involved in presenting antigens to the immune system. Variations in these genes can affect how the immune system recognizes and responds to vaccine antigens. For instance, specific HLA alleles have been associated with vaccine response in diseases like hepatitis B and influenza. Fc receptors are proteins that play a role in the binding and activation of immune cells, such as macrophages and natural killer cells. Genetic variations in Fc receptor genes can impact the binding affinity of antibodies, affecting their ability to clear pathogens and mount an effective immune response. These variations have been linked to the response to vaccines like the influenza vaccine and the hepatitis B vaccine. Furthermore, genetic variations in cytokine genes can influence the production and activity of cytokines, which, in turn, can affect the immune response to vaccines. Polymorphisms in genes such as interleukin-10 (IL-10) and tumor necrosis factor-alpha (TNF-α) have been associated with vaccine response variations. Toll-like receptors (TLRs) play a crucial role in recognizing pathogen-associated molecular patterns (PAMPs) and initiating immune responses. As key pattern recognition receptors (PRRs), TLRs help bridge innate and adaptive immunity and are frequent molecular targets of biomimetic vaccine platforms. At the molecular and cellular levels, these platforms are often engineered to engage TLRs—such as TLR4 via lipopolysaccharide-mimetic ligands or TLR9 via CpG motifs—to enhance dendritic cell (DC) maturation and promote their migration to lymph nodes. Activated DCs present antigens via both MHC class I and II pathways, enabling the activation of CD8+ cytotoxic T lymphocytes and CD4+ helper T cells, respectively. In addition, biomimetic systems may present structural features—such as particulate architectures or repetitive antigen arrays—that enhance B-cell receptor clustering, thereby boosting germinal center formation and the generation of long-lived plasma cells [82].

Importantly, genetic variability in immune-related genes can modulate the effectiveness of these biomimetic interventions. For instance, polymorphisms in TLR genes have been associated with differences in vaccine responsiveness to hepatitis B, measles, and tuberculosis, potentially influencing receptor sensitivity and downstream signaling. Similarly, variations in the CYP2D6 gene, which encodes an enzyme involved in drug and vaccine metabolism, can affect the pharmacokinetics and efficacy of vaccines such as the measles-mumps-rubella (MMR) vaccine. These findings highlight the importance of personalized vaccinology, where host genetic factors are considered in the design and evaluation of biomimetic strategies. Together, the integration of immune engineering and genetic insights offers a powerful path toward more precise and effective vaccination platforms.

### 4.4. Waning Immunity over Time

Waning immunity refers to the gradual decrease in the strength and effectiveness of the immune response over time following vaccination. While vaccines are designed to stimulate a robust and durable immune response, it is common for the intensity of the immune response to decline gradually. One factor is the natural decay of immune memory over time. After vaccination, the immune system forms memory cells that “remember” the specific pathogen and can mount a rapid and targeted response upon re-exposure. However, over time, the number and functionality of these memory cells can decline, leading to a weakened immune response. This decay of immune memory can result in reduced protection against the pathogen [83].

The timing and necessity of booster doses paradoxically vary depending on the characteristics of the pathogen, the vaccine formulation, and the individual’s immune response. Thus, ongoing research and surveillance are essential to determine the optimal timing for boosters and to ensure long-term protection against infectious diseases. To clarify it: Booster doses play a critical role in sustaining vaccine-induced immunity, particularly in the context of waning protection and the continuous emergence of viral variants. Following primary immunization, the immune response naturally contracts over time, and the levels of neutralizing antibodies and memory cells may decline, especially in older adults or immunocompromised individuals. Booster administration serves to reactivate immune memory and significantly increase antibody titers, restoring robust protection and improving the breadth of immune recognition.

This is particularly relevant in the case of rapidly evolving pathogens, such as influenza viruses or coronaviruses, where antigenic drift can reduce the effectiveness of the original vaccine strain. Booster doses help reorient the immune system toward updated antigenic targets, enhancing cross-reactivity and enabling faster recall responses upon exposure. Evidence from COVID-19 vaccination campaigns, for instance, has demonstrated that timely booster doses not only restore immunity but also expand the response to include variants with partial immune escape.

From a biomimetic perspective, the design of next-generation boosters could benefit from systems that mimic sustained or pulsed antigen exposure, similar to natural infection or persistent low-level stimulation. Nanoparticle-based delivery platforms and bioinspired adjuvants can help create longer-lasting and more targeted memory responses, potentially reducing the frequency of booster requirements. Additionally, booster strategies that incorporate mucosal delivery routes may improve protection at the site of pathogen entry, especially for respiratory and enteric viruses. Recommendations:

1. Personalized booster schedules should be developed based on age, immunological status, and exposure risk, moving beyond the one-size-fits-all approach.
2. Surveillance-guided updates of vaccine antigens should be prioritized to match circulating variants, particularly for mutable viruses.
3. Mucosal and microbiota-informed boosters offer a promising frontier and should be further explored for their potential to enhance both systemic and local immunity.
4. Global equity in booster access must be addressed, ensuring that low- and middle-income countries are not left behind in periodic immunization campaigns.

In conclusion, booster doses are not merely repeat administrations but are a strategic component of dynamic immunization programs. They are essential to maintaining long-term protection and adapting to the evolving landscape of infectious diseases, especially when informed by principles of biomimicry and personalized immunology.

## 5. Variability in Vaccine Responses

Variability in vaccine responses is a complex phenomenon that encompasses individual differences in the immune system’s reaction to vaccination. Understanding the factors that contribute to this variability is crucial for predicting vaccine efficacy and optimizing immunization strategies. While characteristics of the pathogen, vaccine formulation, and individual immune response play significant roles, genetic variations and environmental factors also influence vaccine responses [82].

Genetic variations in genes related to the immune system, including human leukocyte antigens, cytokines, and toll-like receptors, can impact how the immune system recognizes and responds to vaccine antigens. These variations contribute to differences in antibody production, cellular immune responses, and overall vaccine effectiveness. Additionally, host genetic factors can influence vaccine metabolism and affect the kinetics and magnitude of the immune response. Environmental factors, such as previous exposure to the pathogen, underlying health conditions, and lifestyle choices, also impact vaccine responses [84]. Pre-existing immunity from previous infections or vaccinations can shape the immune response to subsequent vaccinations, while factors like nutritional status, chronic diseases, and medications can influence immune status and vaccine responses [85]. The timing and spacing of vaccine doses, as well as the use of adjuvants, also play a role in shaping the immune response and subsequent protection.

Yet, a growing focus is being placed on the role of the human gut microbiome in vaccine responses. Recent reviews have highlighted the impact of the gut microbiota on early life immunity, with specific attention given to two key members: Bifidobacteria and Bacteroides. These beneficial microbes have demonstrated health-promoting and immunomodulatory properties, enhancing immune cell maturation, promoting anti-inflammatory molecule production, and maintaining immune balance [86]. Studies have shown that the composition of the gut microbiome can influence vaccine responses, potentially affecting vaccine effectiveness in infants [87]. Modulating the gut microbiota through targeted interventions like probiotics or prebiotics may enhance vaccine outcomes in this vulnerable population [88]. Furthermore, ongoing research is also focused on identifying novel components derived from microbes that can serve as vaccine adjuvants or contribute to the development of safer and more effective vaccines. Exploiting the immunomodulatory properties of specific gut bacteria and their metabolites holds promise for improving vaccine responses and reducing the burden of infectious diseases.

### The Impact of the Infant Gastrointestinal Tract Microbiome on Immunity and Vaccination

Studies have shown that the presence of specific bacterial strains in the infant gut microbiome is associated with enhanced immune responses to vaccines. For example, a high abundance of certain Bifidobacterium strains, such as Bifidobacterium longum subspecies infantis and Bifidobacterium breve, has been linked to increased antibody responses following vaccination against diseases like BCG, tetanus, hepatitis B, and polio [89]. These strains have demonstrated immune-modulating capabilities, promoting the maturation of immune cells and stimulating the production of key cytokines. The mechanisms underlying the influence of the gut microbiome on vaccine responses are multifaceted. One important aspect is the interaction between gut bacteria and the host immune system. Commensal bacteria, such as Bifidobacteria and other species like Bacteroides, produce metabolites like short-chain fatty acids (SCFAs) through the fermentation of dietary fibers. SCFAs interact with immune-cell receptors and help regulate immune responses [90], potentially enhancing vaccine efficacy.

Furthermore, the gut microbiome can influence the development and function of various immune cells. Regulatory T cells (Treg) and B lymphocytes, important components of the immune system, are influenced by specific bacterial strains like Bifidobacterium pseudocatenulatum and Bifidobacterium bifidum [91]. These strains have been found to restore a balanced state of Treg and B lymphocytes, thereby reducing systemic inflammation and promoting immune homeostasis.

Dendritic cells, which are responsible for initiating immune responses, can also be influenced by gut bacteria [92]. Bifidobacterium bifidum strains have been shown to stimulate dendritic cells and induce specific immune profiles, such as Th17 responses, which can further enhance the differentiation of Treg cells and modulate immune function [93]. Additionally, certain Bifidobacterium strains have been found to increase the cytotoxic activity of CD8+ T cells, without affecting CD4+ T-cell activity [94].

However, the impact of the gut microbiome on vaccine responses is complex and can also exhibit variability. Studies have reported contradictory findings regarding the association between specific bacterial phyla, such as Bacteroidetes, and vaccine responsiveness. Some studies have shown a negative correlation between Bacteroidetes prevalence and vaccine response [95], while others have observed increased concentrations of this phylum in vaccine responders [96]. These discrepancies highlight the need for further research and larger-scale studies to better understand the role of specific bacterial species and strains in vaccine variability.

The variability in vaccine responses may also be influenced by factors such as the timing of microbial colonization, host genetics, environmental exposures, and other individual-specific factors [97]. The intricate interplay between the gut microbiome, immune system, and vaccines necessitates a comprehensive approach to optimize immunization strategies. Developing a deeper understanding of the specific mechanisms by which gut bacteria influence vaccine responses will enable the development of targeted interventions, such as prebiotic or probiotic supplementation, to enhance vaccine efficacy.

Furthermore, innovative approaches using whole bacteria or their products and metabolites have shown potential, as observed in the augmentation of immune checkpoint inhibitor responses in cancer treatments [98,99,100]. This opens the possibility for preclinical studies to quickly progress to clinical trials, particularly considering the generally recognized safety of certain Bifidobacterium species.

A more targeted approach involves focusing on specific microbial products, such as bacterial extracellular vesicles (BEVs) [101]. The success of BEV vaccines against diseases like cholera and type B meningitis, as well as the promising results with commensal BEV antigen carriers against plague and influenza, suggests that a new generation of vaccines based on immunomodulatory BEVs from commensal sources could be developed with high efficacy and biosafety standards on a global scale [18]. However, further research is needed to identify strains that produce novel adjuvant-like BEVs from early-life microbiota, including Bifidobacteria and Bacteroides.

## 6. Impact of Vaccination on Transmission

The impact of vaccination on transmission has been traditionally studied [102] since the protection of the vaccine is irregular because it depends on the virus’s antigenic evolution. The influenza vaccine is one of the most widely used all around the world, and it has been proven that annual variation in immunity duration affects the vaccine effectiveness through a raised infection attack rate. As a result, the effectiveness of a vaccine should not be considered in a one-year span, but in the long term, in which the vaccination coverage should be large [103] enough to be analysed.

Not only the vaccine but also the vaccination strategy affects its impact. Mortality-based strategies address high-risk populations, while morbidity-based strategies aim for high-prevalence populations. The best approach depends on the reproductive ability of the virus. Usually, morbidity-based strategies are better when the viral transmission is low, but not for higher reproductive rates of the virus [104]. In any case, it is important that public health agencies collect estimates of the transmission to make a decision, and when not available, address mortality-based strategies as a priority.

In December 2009, the influenza pandemic (H1N1) was assessed to better analyze what characteristics could trigger a third wave. The top three mechanisms were: the environmental conditions and the virus adaptation; the development of a variant that could be harmless to the vaccine; and changes in socialization during school holidays. These factors should be considered within the vaccination strategy to mitigate subsequent pandemic waves [105].

The importance of younger people transmitting influenza when there are school holidays seems to be a matter of concern among researchers. Older age groups usually get infected within households in familiar settings, above all through asymptomatic individuals. These insights also highlight the vulnerability of the elderly due to close contact with younger asymptomatic caregivers. Apparently, morbidity-based strategies tend to control the transmission process among young people (more active) than mortality-based strategies, which target very young children and very old people, who are relatively inactive, thus transmitting the virus at a much lower rate [106].

Children aged 2–18 seem to be key contributors to influenza transmission. Previous research in England and Wales suggests that a 50% vaccination rate of children between 2 and 18 years old could lead to a significant decrease in influenza-related morbidity and mortality for the population, including the elderly and other high-risk groups, which also had to be prioritized in the vaccination strategy. Apparently, the paediatric vaccination strategy has the potential to fight the clinical burden that influenza causes in health care centers [107].

Several strategies can model the impact of vaccines on transmission. Among them, a Bayesian modeling approach, together with a simple dynamical model, was used in the United States to better understand how the influenza vaccine affects transmission. With around 45% of the population vaccinated, the reduction in transmission varied between 20.8% and 47.5%. However, these results decreased to only 1.04% for the H1N1 pandemic, in which the vaccination development was late. Therefore, vaccination coverage and early development of a vaccination strategy to protect the most vulnerable populations, like the elderly, seem to be crucial [108].

In recent studies conducted among Danish households, the SARS-CoV-2 Delta variant, known for its higher transmission rates compared to previous variants, was examined. The research revealed that the effectiveness of the vaccine in preventing transmission was 42%. Furthermore, it was observed that unvaccinated individuals had an increased viral load in comparison to fully vaccinated individuals. To evaluate the vaccine effectiveness against susceptibility to infection and vaccine effectiveness against infectiousness, the authors included 24,693 households with a primary case of SARS-CoV-2 infection, specifically focusing on the Delta Variant of Concern in 2021. This encompassed a total of 53,584 household contacts. Authors showed that unvaccinated secondary cases with an infection exhibited a threefold higher viral load compared to fully vaccinated secondary cases with a breakthrough infection. This highlights the role of vaccination in not only reducing the likelihood of infection but also lowering viral replication and shedding among breakthrough cases, potentially reducing the risk of transmission [109].

The Omicron variant was soon predominant after Delta, and its differences in transmission were assessed in England through logistic regression models. The transmission was lower in those who had three SARS-CoV-2 vaccines when compared to those who had only two. The vaccines were less effective in stopping the transmission of the new variant Omicron when compared to Delta, which suggests an evolution of the virus to lower its harm and increase its transmission [110].

### Real World Scenarios

Real-world immunization experiences provide critical insights for the refinement of biomimetic vaccine strategies. For instance, the co-administration of Bacillus Calmette–Guérin (BCG) and diphtheria–tetanus–pertussis (DTP) vaccines has demonstrated variable immunological outcomes depending on sequence and timing, highlighting the importance of context-specific scheduling. The global COVID-19 vaccination campaign further underscored the role of booster doses in maintaining protection across emerging SARS-CoV-2 variants, revealing the need for adaptive strategies and real-time monitoring. Additionally, during the 2009 H1N1 influenza pandemic, prioritization frameworks based on age, comorbidity, and exposure risk illustrated the challenges and ethical dimensions of vaccine distribution in emergency settings. These cases exemplify the dynamic interface between immunological theory, population health, and practical deployment constraints.

Thus, Vaccination remains the most effective way to avoid the propagation of viruses, and not only its efficiency but also the strategy to follow to protect high-risk groups and high-transmission groups are the key facts to stop the transmission of the virus and to decrease the risk of severe symptoms. There is a need to keep improving the vaccines to contain pandemics, with more long-term effects and better communication strategies to convince the population about the need to tackle extreme diseases [111].

## 7. Vaccine Effectiveness Against Emerging Variants

Vaccine effectiveness against emerging variants is a crucial area of research and consideration for infectious disease control. While the focus on COVID-19 variants has been prominent, the concern for emerging variants extends to other infectious diseases as well. Pathogens such as influenza, HIV, and malaria have demonstrated a propensity for genetic mutations that give rise to new strains with altered antigenic profiles. These changes can impact the ability of existing vaccines to provide adequate protection. Monitoring the effectiveness of vaccines against these evolving strains is essential to understand whether updated formulations or alternative vaccination strategies are needed. Research efforts encompass epidemiological studies, in vitro assays, and animal models to evaluate the neutralizing capacity of existing vaccines against variant strains. Furthermore, understanding the underlying mechanisms of vaccine breakthrough infections with emerging variants will aid in designing more effective and adaptable vaccines for future outbreaks and pandemics. Ensuring vaccine effectiveness against emerging variants is a critical aspect of global health preparedness and safeguarding public health [110,111,112,113,114].

The intrahost variability among virus populations is a key factor regarding the evolution of each strain and the harm it can cause in the future. This issue is crucial for public health, and one of the most important factors to understand is the capacity of vaccines in dealing with a virus sample diversity [112].

Influenza vaccines have been traditionally designed to consider the virus variability over the years [113]. Every year, there is an antigenic drift able to survive from the last year’s vaccine, and this fact ultimately depends on the host [114]. Previous research has studied viruses obtained from patients participating in a vaccine efficacy trial, showing that most of the genetic variations within hosts were infrequent and only a small number of variants were shared among participants. The level of intrahost diversity did not present a significant variability based on preseason antibody levels. Opposite to previous research in this field, this study showed that seasonal influenza vaccination has a limited effect on intrahost diversity during natural infection [114].

The SARS-CoV-2 pandemic was a huge challenge to the public health systems due to its rapid propagation and mutation into different variants. Variants B.1.1.7 and B.1.351 were reported to become especially infectious and able to resist vaccines [115,116]. Experts highlighted the importance of increasing the number of doses to enhance the immune response, together with boosting the development of new-generation vaccines to adapt to new variants and mutations [116,117].

In the United Kingdom, the effectiveness of the SARS-CoV-2 vaccines (BNT162b2 and ChAdOx1) against the new B.1.617.2 (Delta) variant. They used surveys in randomly selected households and found that the effectiveness of each vaccine was reduced up to 13% (BNT162b2) and 16% (ChAdOx1). They also reported that two doses of the vaccine were at least as protective as a previous natural infection. Both vaccination and the combination of vaccination and natural infection were especially positive in younger adults in resisting the infection, although a peak viral burden was a key factor in getting infected with the Delta variant [118]. Clinical trials about the SARS-CoV-2 vaccine reported it to be effective against the first strain of COVID-19 (95,6%), and protective with less percentage success with the new variants B.1.1.7 (85,6%), and B.1.351 (60%) [119].

A similar study was conducted in Southern California, in which two doses of the Moderna COVID-19 vaccine were effective against all SARS-CoV-2 variants (including Delta), especially to avoid hospitalization. They studied 8153 cases and controls, reporting an effectiveness of 86.7% against the Delta variant and 98.4% against the Alpha variant. However, there was a reported lower power to combat infection with Delta as time passed by since vaccination (from 94.1% 14–60 days after vaccination to 80% 151–180 days after vaccination) [120].

In Brazil, 11,817 PCR tests were analyzed to assess the effectiveness of the Ad26.COV2.S (Janssen, Tokyo, Japan) against COVID-19. They found a 50.9% effectiveness against symptoms after 28 days of the first dose, 72.9% against hospitalization, 92.5% against Intensive Care Unit admission, and 90.5% against death. These results reinforced the power of a single dose against COVID-19 symptoms in a period in which new variants were emerging [121].

In India, the efficacy of a booster dose and the performance after a two or three-dose vaccine in a randomized controlled trial. After 12 months of vaccination, there was a decrease in the magnitude of antibodies, but there was a persistence of immunity. The third dose was particularly effective in neutralizing different variants and strains like Alpha, Beta, Delta, and Omicron, and these results showed the importance of boost doses to fight against new strains and variants of SARS-CoV-2 [122].

In Canada, the effectiveness of a single-dose mRNA vaccine was studied among a high-risk population of 70 years and older. Among the 16,993 specimens analysed, they obtained vaccine effectiveness of 72% for variants different from Alpha and Gamma, 67% for the Alpha variant, and 61% for the Gamma variant. These results showed how a mRNA vaccine could reduce the risk of SARS-CoV-2 infection, including the variants Alpha and Gamma, which had only a minimal reduction of their protection when compared to the previous ones [123].

In South Africa, 4387 adults (18–84 years) were randomized and dosed with either NVX-CoV2373 or placebo, with an efficacy of 49,4%, and 60.1% among HIV-negative participants. The efficiency of the vaccine against the B.1.351 variant found in South Africa was 51.0% in HIV-negative adults, with mild to moderate symptoms primarily due to this new variant [124].

One of the most important problems to stop the virus transmission is its mutation in the viral spike protein, which could neutralize antibodies after it alters the virus-host cell interplay. These kinds of mutations can enhance the capacity to avoid vaccine responses [125,126,127].

Vaccination helps to combat new variants of viruses, and it fosters a better immune response to better prepare for new mutations of each strain. Rapid research and development of new vaccines to tackle new variants are crucial, but a key point in the strategy to combat new strain transmission is for the population to be vaccinated as soon as possible with a dose that can combat the original version of a virus, at least to prevent severe disease (Table 1). To be effective, the vaccine coverage should consider the importance of robust memory response and acceptance among the population [111].

## 8. Immunosenescence and Vaccination in Older Adults

Immunosenescence refers to the gradual decline in immune function that occurs with aging, leading to increased susceptibility to infections and decreased response to vaccinations in older adults [128]. Understanding age-related changes in the immune system is crucial to optimizing vaccine immunogenicity and effectiveness in this population. Several alterations in the immune system contribute to immunosenescence, including reduced production of naïve T cells, impaired B cell function, and dysregulation of innate immune responses [129]. These changes result in a decline in the ability of the immune system to mount a robust response to vaccines, leading to lower antibody production and decreased protection against infectious diseases.

To overcome the challenges posed by immunosenescence, various strategies have been explored to enhance vaccine responses in older adults. One approach is the use of high-dose vaccines, which contain a higher concentration of antigens to stimulate a stronger immune response [130]. Another strategy involves the use of adjuvanted vaccines, which incorporate substances that enhance the immune response, such as MF59 and AS01 (Turley, J. L., & Lavelle, E. C. 2022) [131]. These adjuvanted vaccines have been shown to improve immunogenicity and vaccine efficacy in older adults.

In this line, when addressing immunosenescence and vaccination in older adults, various strategies have been explored to improve vaccine responses. These include the use of novel vaccine formulations, immune modulation approaches, targeted vaccination strategies, personalized vaccination approaches based on individual immune profiles, and the utilization of vaccine adjuvants. These strategies aim to enhance immunogenicity and effectiveness in older adults by considering age-related changes in the immune system. While further research and clinical trials are needed to validate their efficacy and safety, these strategies offer promising avenues to optimize vaccine responses and address the challenges posed by immunosenescence in older adults [130,132,133].

Additionally, the timing and scheduling of vaccinations play a crucial role in optimizing immune responses in older adults. Studies have shown that providing vaccines earlier in the day, when immune responses tend to be stronger, can result in improved vaccine effectiveness [129,131]. Furthermore, spacing out vaccine doses appropriately and ensuring adequate intervals between vaccinations can help maximize the immune response. It is worth noting that personalized approaches to vaccination based on individual immune profiles may hold potential in improving vaccine responses in older adults. Identifying biomarkers of immune function and tailoring vaccine strategies accordingly could lead to more targeted and effective immunization strategies [134].

In addition to exploring age-related changes in the immune system and strategies to enhance vaccine responses, it is crucial to consider the broader context of immunosenescence and vaccination in older adults. One aspect to consider is the importance of promoting vaccine awareness and education among older adults. Many individuals may not be fully aware of the benefits of vaccination or the specific vaccines recommended for their age group. Implementing targeted educational campaigns and providing accessible information can help increase vaccine uptake and adherence among older adults [6]. Furthermore, healthcare providers play a vital role in advocating for and administering vaccines to older adults. It is essential for healthcare professionals to stay updated on current vaccination guidelines, engage in effective communication with their patients, and address any concerns or misconceptions regarding vaccines [135].

Collaboration between healthcare providers, public health agencies, and community organizations is also crucial in creating effective vaccination programs for older adults. By working together, these stakeholders can develop strategies to reach underserved populations, provide convenient access to vaccines, and implement targeted interventions to address barriers to vaccination [136]. Lastly, ongoing research and surveillance are essential in monitoring vaccine effectiveness and identifying potential gaps in protection among older adults. This can help guide the development of new vaccines, refine existing vaccination strategies, and ensure that older adults receive the most appropriate and effective vaccines for their age group [137].

In conclusion, understanding the impact of immunosenescence on vaccine responses is crucial for developing effective vaccination strategies for older adults. Further research and development of vaccines that specifically target the age-related changes in the immune system are needed to ensure optimal protection against infectious diseases in this vulnerable population.

## 9. Maternal Vaccination and Neonatal Immunity

Maternal immunisation is considered a useful tool in public health promotion, as it can protect both the mother and her foetus against various infectious diseases. In this line, the World Health Organisation (WHO) describes how maternal infections are estimated to be responsible for 10 to 50% of fetal death, as well as to contribute to 25% of neonatal deaths [138]. Thus, vaccination of pregnant women in the second or third trimester induces the production of specific antibodies that could cross the placenta and subsequently, may protect the fetus. Hence, it has been described by previous researchers how the transport of immunoglobulin G (IgG) may be detected at about 17 weeks of gestation, reaching higher values in fetal serum than in maternal serum at 40 weeks of gestation. Furthermore, similar outcomes were found regarding breastfeeding, where raised IgG values were found in breastmilk [139]. These findings suggest how maternal vaccination may enhance fetal and newborn immunity, being an important target in order to safeguard the infant’s integrity.

Different institutions proposed the utilisation of different vaccines with the aim of reducing infectious diseases in children. In this line, the WHO proposed to achieve Maternal and Neonatal Tetanus Elimination (MNTE) through the maternal immunisation of tetanus [140]. Vaccination yielded encouraging results, as the number of developing countries that have not yet achieved tetanus elimination has been reduced from 59 countries in 2000 to only 12 in July 2019 [141]. Regarding influenza maternal vaccination, it has been proposed by the WHO during the influenza period since 2005 [142]. Nevertheless, this recommendation was avoided for several years, until the H1N1/09 influenza pandemic began, with maternal vaccination against seasonal influenza being promoted in 2010 [143]. Furthermore, national programs, as developed in the Department of Health in the United Kingdom as well as in the Centers for Disease Control and Prevention (CDC) in the United States, proposed the combined immunisation against different pathogens, including pertussis, tetanus, diphtheria, polio and seasonal influenza [144,145].

Regarding pertussis, the previous literature proposed how pertussis antibodies also may cross the placenta, reach the fetus and protect it [146]. Moreover, following an outbreak of whooping cough deaths in 2012, in which 14 children died because they were too young to be vaccinated, the UK’s Joint Committee on Vaccination and Immunisation promoted the vaccination of pregnant women in their 28 through 38 weeks of pregnancy, in order to avoid children’s infection, as they would be passively immunised since pregnancy. This program also involved the administration of low-dose diphtheria and inactivated poliomyelitis vaccine [147,148]. As a result, the incidence of pertussis in children younger than 3 months decreased from 234 cases per 100,000 in 2012 to 52 cases per 100,000 in 2019 [149]. In the US, the CDC suggested the vaccination of tetanus, diphtheria, and acellular pertussis in pregnant women during weeks 27 through 36 of gestation [144]. Concerning the safety of these vaccines, it was highlighted by previous literature how pertussis vaccination in pregnancy was harmless both in mother and fetus, since researchers did not find evidence of a raised maternal or neonatal death risk, nor cardiovascular alterations such as eclampsia or preeclampsia. Besides, no higher risk was found in mothers regarding haemorrhage, uterine rupture, placenta or vasa praevia, and no increased risk was found in fetuses regarding fetal distress, low birth weight or neonatal kidney failure [147]. Similar results were found analysing the immunogenicity of tetanus toxoid and reduced diphtheria toxoid acellular pertussis, where no adverse events were found in pregnant vaccinated women or infants [150].

Regarding other vaccines which may also be contemplated in pregnancy, hepatitis B and meningococcal immunisation have also been pointed out, depending on different situations [145]. In case of hepatitis B, the previous literature suggested how vaccination during pregnancy should be taken into account if the mother presents any risk factors such as have not been vaccinated previously become pregnant, have one or more sexual contacts in the last six months, have knowledge of a household contact or a Hepatitis B positive sexual contact, or intravenous drug utilisation [151]. In case of meningococcal disease, vaccination should be considered for pregnant women if they present associated risk factors which may predispose to Neisseria meningitidis growth, such as living in close contact, suffering complement deficiencies or asplenia, or living in hyperendemic areas [145]. In this line, research conducted in the third trimester of pregnant women who were vaccinated with the meningococcal tetravalent polysaccharide vaccine highlighted how patients showed a suitable maternal antibody transplacental crossing, whereas a speedy diminishing immunity occurred in 3-month-old infants [152]. As an advantage, the previous literature has shown the safety of the vaccine, as no association was found between teratogenesis, spontaneous abortion or preterm birth in women who received meningococcal tetravalent polysaccharide vaccine [153].

In terms of COVID-19 vaccination in pregnant women, controversial outcomes are present in the literature, since the lack of safety data in pregnancy may compromise this decision. Then, the Joint Committee on Vaccination and Immunisation recently proposed that pregnant women encounter the condition of being really vulnerable to COVID-19 and/or suffer clinical conditions, such as organ transplants or consuming immunosuppressant drugs, chronic kidney disease, cystic fibrosis, asthma or cardiovascular disease, which may predispose them to a worse prognosis in COVID-19 disease, may discuss with her obstetrician the possibility of COVID-19 vaccination [154]. Regarding vaccines under research, recent investigators put their efforts into the group B streptococcus vaccine and the development of respiratory syncytial virus vaccines. Group B streptococcus is one of the principal sources of neonatal sepsis and meningitis [155], and its vaccination has been proposed as a useful tool in order to prevent both early and late onset disease in newborns [156]. In this line, a multivalent conjugated group B streptococcus vaccine is currently in phase I and II trials in pregnant women with the aim of establishing if a passive immunity may exist in their young infants [157]. Concerning the respiratory syncytial virus vaccine, the newest literature proposed that a bivalent respiratory syncytial virus prefusion F protein-based vaccine administered to pregnant women was effective in severe respiratory syncytial virus illness in infants, and no adverse effects were found [158].

## 10. Impact of Co-Administration of Vaccines

A combined vaccine could be defined as a vaccine where two or more antigens exist, and it could be presented as ready to use, or it could be presented in a pharmaceutical form, which allows proximate mixing prior to administration. Furthermore, it also could involve the administration of different vaccines on the same day, sited in different anatomic locations. The aim of this process is to provide immunization against several strains of infectious microorganisms or numerous infectious diseases that cause similar illnesses [159].

Co-administration of vaccines has been proposed as an advantage in public health systems, as it facilitates compliance with the vaccination schedule and promotes the use of accelerated vaccination strategies. Contrary to what is widely believed, co-administration has been proposed as a safe tool in order to improve vaccination guidelines, and it shows a comparable reactogenicity profile to those vaccines that are administered alone [160].

In this line, it has been proposed that co-administration may have positive effects on several immunisation outcomes. This is the case of the co-administration of bacille Calmette-Guerin (BCG) and diphtheria-tetanus-pertussis (DTP), which has shown encouraging results in mortality decrease [161]. The study aims to compare the reduction in mortality when both vaccines are administered at the same time, compared with WHO recommendations, which suggest BCG vaccination first, followed by DTP vaccination. Then, researchers reanalysed data from an immunisation conducted in children born and vaccinated in MATLAB (14.2), Bangladesh, between 1986 and 1999. This study proposed that mortality was lower in combined vaccination, with 16/1000 cases reported, compared to 32/1000 cases in children who received BCG-first or 20/1000 cases in those who were vaccinated with DTP first. Similar results were found in other areas, such as in Maharashtra, India, where co-administration showed reduced mortality outcomes, with the mortality rate ratio 0.15 (0.03–0.70) [162]. Additionally, in a study conducted in Senegal, where 73% of the children were co-vaccinated with both BCG and DTP, participants presented fewer mortality than those who were vaccinated with BCG or DTP first [163]. These findings suggest that a potential mechanism of action may exist when a combined vaccination is developed. Nevertheless, the immunological effect of co-administering BCG and DTP has not been elucidated yet. However, the recent literature proposed that BCG may be related to epigenetic modifications, which may reschedule monocytes’ activity to nonspecific protection against unrelated infectious diseases [164,165].

Concerning other types of vaccines, such as the meningococcal C vaccine, the recent literature proposed the advantages of the co-administration of it combined with measles, mumps, rubella, and varicella (MMRV) vaccine in children over 12 months of age [166,167,168]. Thus, Vesikari et al. proposed that the co-administration of MMRV and meningococcal C vaccines would produce similar results in immunogenicity or safety profiles as in single vaccination [166]. Likewise, a few years later, Scott et al. proposed how co-administration of the MMRV vaccine combined with meningococcal C yielded a strong immune response to all strains of both inoculations [169]. Moreover, numerous researchers suggested that the appearance of side effects was similar in single vaccination and co-administration of both vaccines [166,167], as well as the simultaneous administration presented an optimal tolerance and absence of safety complications [167]. Concerning the efficacy issues, a study conducted in Italy involving 716 children proposed that the antibody titers against MMRV antigens, detected by enzyme-linked immunosorbent assays (ELISA), showed the same protective levels when MMRV was simultaneously administered with meningococcal C vaccine or when it was administered alone [170]. Similar results were found regarding other types of vaccines co-administered with meningococcal C strains, such as seasonal influenza vaccine, hepatitis A and B vaccine, as well as the DTaP-HepB-IPV/Hib vaccine. Regarding co-administration with seasonal influenza vaccine, in a study conducted in the Philippines, meningococcal and influenza simultaneous inoculation showed an adequate safety profile in adults, with the most common adverse effects being pain and headache. Concerning the efficacy, it was determined that no significant differences in vaccine response terms were found between those adults immunised with meningococcal and influenza vaccines and those meningococcal inoculated alone. Nevertheless, lower antibody titers and lower levels were found in the co-administration groups compared to the single meningococcal inoculation groups. These results suggest the probability of some immune interference between both microorganisms [171]. Regarding Hepatitis A and B, the previous literature highlighted that the co-administration of the meningococcal vaccine and Hepatitis A and B vaccines presented an optimal safety and efficacy profile. Thus, these researchers pointed out how 609 adolescents showed adequate meningococcal antibody titer from 1 to 7 months post vaccination, regardless of whether they were inoculated with meningococcal alone or co-administered with both hepatitis vaccines [172]. Finally, concerning the simultaneous administration of meningococcal and DTaP-HepB-IPV/Hib vaccine, it was proposed that the co-administration involved an acceptable safety profile as all groups presented similar reactogenicity. Additionally, it was shown that administration of both vaccines together was not lower than administration of either vaccine separately. Moreover, meningococcal antibody titers were satisfactory, and there were no significant differences regarding hepatitis B, Haemophilus influenzae b, diphtheria, tetanus, and polio immunogenicity [173].

Regarding adults’ vaccination and as a consequence of the COVID-19 pandemic, several researchers proposed the possibility of vaccinating against both the influenza virus and COVID-19. Concerning potential advantages, combined COVID-19 and seasonal influenza vaccination has shown numerous benefits, such as enhanced patient convenience and agreement, simplified vaccination guidelines, lower missed opportunities to vaccinate, as well as a reduction in costs [174,175,176,177,178]. Then, special population groups have been proposed as the primary targets in co-administration of COVID-19 and seasonal influenza virus vaccines, including elderly patients as well as at-risk adults, who can benefit from these advantages [179,180]. Moreover, important agencies such as the WHO and the CDC suggested that COVID-19 vaccines may be co-administered with other immunisation tools, including seasonal influenza virus [181,182]. Nevertheless, controversial results have been found regarding this co-administration. Some of them found a negative association between seasonal influenza virus reporting rates and SARS-CoV-2-related results, such as death [183,184]. On the contrary, protective outcomes were found in other studies [185,186,187], whereas other authors proposed that there is no association between the co-administration of both vaccines [188,189]. Moreover, in order to clarify conclusions, both a systematic review and meta-analysis of this non-specific association were developed [190], showing a significant decrease in positive laboratory-confirmed cases of SARS-CoV-2 in those patients who were vaccinated with the seasonal influenza virus (OR = 0.86; 95% CI: 0.79–0.94). Nevertheless, regarding other outcomes such as hospitalization, admission to intensive care units or mortality, no significant associations were found (Table 2). Regarding safety concerns, no major adverse effects were found in the co-administration of influenza virus and COVID-19 vaccines, as similar results concerning side effects (most of them related to pain in the injection location) were found both in patients co-vaccinated and in the case of those vaccinated only with COVID-19 [191].

## 11. Factors Influencing Vaccine Failure

Vaccines have been hailed as one of the most effective public health interventions in history. They have helped to prevent the spread of infectious diseases and have saved countless lives. However, despite their success, vaccines do not always work as intended. Vaccine failure can occur for a variety of reasons, including improper storage, administration errors, and vaccine hesitancy. In this discussion, we will explore the factors that contribute to vaccine failure and the importance of addressing these factors to improve vaccine effectiveness [192].

Some of the factors contributing to vaccine failure are the improper storage and handling of vaccines, which can lead to a loss of potency and effectiveness. Vaccines require specific storage conditions, including temperature and humidity control, to remain effective. It was found that up to 25% of vaccines may be damaged due to improper storage and handling (WHO, 2020) [193]. This can result in reduced vaccine efficacy and an increased risk of vaccine failure. Another study found that improper storage of vaccines can lead to a significant reduction in vaccine efficacy, with some vaccines losing up to 50% of their potency [194].

Administration errors are another factor that can contribute to vaccine failure. These errors can range from incorrect dosing to improper injection technique. The Centers for Disease Control and Prevention (CDC) found that administration errors were responsible for up to 50% of vaccine failures [182]. This highlights the importance of proper training for healthcare providers who administer vaccines. Another study found that training healthcare providers in proper vaccine administration techniques can significantly reduce the incidence of administration errors [195].

Vaccine hesitancy is a growing concern that can also contribute to vaccine failure. Vaccine hesitancy refers to the reluctance or refusal to vaccinate despite the availability of vaccines. A study published in the journal Vaccine found that vaccine hesitancy was associated with lower vaccination rates and an increased risk of vaccine-preventable diseases [196]. This underscores the need for effective communication and education to address vaccine hesitancy. Another study found that providing parents with accurate information about vaccines can help to increase vaccine acceptance and reduce vaccine hesitancy [197].

Addressing the factors that contribute to vaccine failure is crucial to improving vaccine effectiveness. Proper storage and handling of vaccines can help to ensure that they remain effective and potent. This requires adequate training for healthcare providers and proper storage facilities. A study conducted in India found that implementing proper storage and handling practices for vaccines led to a significant improvement in vaccine efficacy [198]. Reducing administration errors is also essential for improving vaccine effectiveness. This can be achieved through proper training and education for healthcare providers who administer vaccines. Improved communication between healthcare providers and patients can also help to address vaccine hesitancy and improve vaccination rates. A study conducted in Australia found that providing healthcare providers with training in communication skills can help to increase vaccine uptake [199]. Liposome-based delivery systems represent one of the most clinically validated examples of biomimetic nanotechnology in vaccine development. Mimicking natural lipid bilayers, liposomes facilitate the encapsulation and delivery of nucleic acids, proteins, or small molecules. Their role has been pivotal in the success of mRNA-based COVID-19 vaccines (e.g., BNT162b2 and mRNA-1273), enabling stable mRNA transport, cellular uptake, and cytosolic release. The established safety, scalability, and versatility of liposomes make them a cornerstone for future biomimetic vaccine delivery platforms [200].

Vaccine failure can occur for a variety of reasons, including improper storage, administration errors, and vaccine hesitancy. Addressing these factors is essential for improving vaccine effectiveness and reducing the spread of vaccine-preventable diseases. Proper training and education for healthcare providers, improved communication with patients, and adequate storage facilities can all help to address these issues and improve vaccine effectiveness.

## 12. Immunomodulatory Adjuvants and Vaccine Enhancement

Immunomodulatory adjuvants are substances that enhance the immune response to antigens in vaccines. However, the use of these adjuvants may also lead to vaccine enhancement, which is the exacerbation of disease upon subsequent exposure to the pathogen. The mechanisms underlying vaccine enhancement are not fully understood, but it is thought to be related to the dysregulation of the immune response, leading to an overproduction of pro-inflammatory cytokines. Therefore, the development of safe and effective adjuvants that do not cause vaccine enhancement is crucial for vaccine development [201]. One potential approach to achieving this is the use of nanoparticle-based adjuvants. These adjuvants have been shown to enhance the immune response without causing vaccine enhancement in preclinical studies [201]. Another approach is the use of combination adjuvants, which may have a synergistic effect while minimizing the risk of vaccine enhancement [202].

Immunomodulatory adjuvants exert their effects through various mechanisms. They can activate innate immune cells, such as dendritic cells, macrophages, and neutrophils, leading to the secretion of pro-inflammatory cytokines and chemokines. This activation promotes antigen uptake, antigen presentation, and the induction of adaptive immune responses. Additionally, adjuvants can enhance antigen stability, promote antigen depot formation, and modulate the signaling pathways involved in immune cell activation [203]. These mechanisms collectively contribute to the enhanced immunogenicity of vaccines. Immunomodulatory adjuvants can shape both innate and adaptive immune responses. Innate immune cells recognize pathogen-associated molecular patterns (PAMPs) through pattern recognition receptors (PRRs), leading to the production of pro-inflammatory cytokines and the recruitment of immune cells to the site of vaccination [204]. Adjuvants can activate PRRs directly or indirectly, promoting innate immune cell activation and antigen presentation. This, in turn, leads to the activation of antigen-specific T cells and B cells, resulting in the production of effector cells and long-lasting memory responses [205].

Several studies have provided insights into the effects of immunomodulatory adjuvants on innate immune responses, for example, in a study that investigated the role of toll-like receptor (TLR) agonist adjuvants in activating dendritic cells. The researchers demonstrated that the TLR agonist adjuvants promoted the maturation and activation of dendritic cells, leading to enhanced antigen presentation and subsequent T cell activation [206]. In addition to their effects on innate immune cells, immunomodulatory adjuvants also influence adaptive immune responses. Adjuvants can enhance the activation of antigen-specific T cells and B cells, resulting in the production of effector cells and the generation of long-lasting memory responses [207]. The activation of adaptive immune responses is critical for establishing durable protection against pathogens.

Research has provided valuable insights into the impact of adjuvants on adaptive immune responses. For instance, a study aimed to investigate the effects of a nanoparticle-based adjuvant on antigen-specific CD8+ T cell responses. The researchers found that the adjuvant significantly enhanced the expansion and activation of antigen-specific CD8+ T cells, leading to improved cytotoxic T cell responses [208]. Moreover, adjuvants can shape the differentiation of antigen-specific B cells, influencing the production of high-affinity antibodies and the development of long-term humoral immune responses. Another study examined the effects of an alum-based adjuvant on the induction of antigen-specific antibody responses. The researchers demonstrated that the adjuvant enhanced the production of antigen-specific antibodies and promoted the formation of long-lived plasma cells [209]. These studies highlight the diverse effects of immunomodulatory adjuvants on both innate and adaptive immune responses. By activating innate immune cells and promoting antigen-specific T and B cell responses, adjuvants enhance the immunogenicity of vaccines and contribute to the development of robust and long-lasting protective immunity.

Despite their benefits, the development of immunomodulatory adjuvants faces several challenges. Safety considerations, such as the potential for adverse reactions or systemic inflammation, must be carefully addressed [210]. Additionally, adjuvant selection requires a balance between inducing robust immune responses while avoiding excessive reactogenicity [211]. Future research should focus on identifying novel adjuvants with improved safety profiles and enhancing our understanding of the mechanisms underlying adjuvant-mediated immune enhancement. Furthermore, the development of adjuvants that can specifically target certain immune cell subsets or modulate immune responses in specific disease contexts holds promise for personalized vaccination strategies [212].

Immunomodulatory adjuvants have revolutionized vaccine development by enhancing immunogenicity and promoting protective immune responses. Their ability to modulate innate and adaptive immune responses has paved the way for the development of effective vaccines against challenging pathogens. Ongoing research and future investigations are necessary to identify new adjuvants, optimize their formulation, and understand their mechanisms of action fully. By harnessing the potential of immunomodulatory adjuvants, we can continue to advance vaccine design and contribute to global health.

## 13. Assessing Vaccine Effectiveness in Real-World Settings

**Figure 3:**
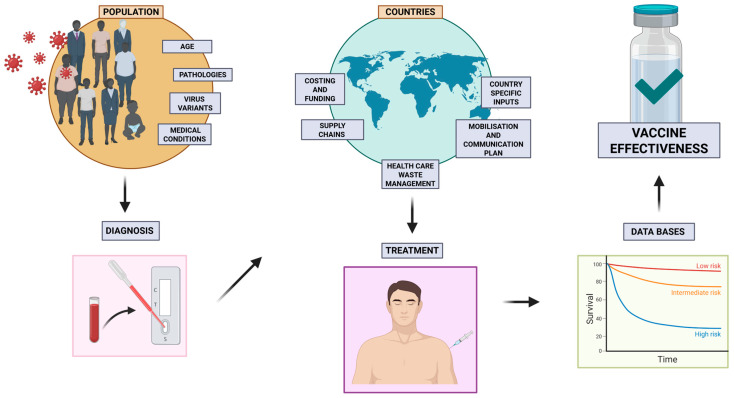
Preliminary procedures that take place before the effectiveness of a vaccine can be determined, ranging from the characteristics of the population to the resources per country and the distribution of the vaccine, such as the data collection process.

Comparing the frequency of health outcomes in vaccinated and unvaccinated individuals is a common method for determining vaccine effectiveness (VE). Estimating the efficacy of vaccines released to the public in real-world conditions is crucial since the settings of clinical trials may have an impact on their results [213]. Host-related factors, such as age, presence of underlying medical conditions (e.g., diabetes, cancer), and history of prior infection; pathogen-related factors, such as the virus variant(s) circulating; and vaccine-related factors, such as vaccine type and time since vaccination, all contribute to vaccine efficacy (Figure 3) [70].

A number of vaccinations have been evaluated and given permission for emergency use since the COVID-19 pandemic. The ChAdOx1 nCoV-19 vaccine (AZD1222; Oxford-AstraZeneca) demonstrated excellent VE against severe acute respiratory syndrome coronavirus 2 (SARS-CoV-2) infection in phase III studies [214]. Also, other studies verifying efficacy in a total of 5948 COVID-19-related hospitalisations, of which 1245 (21%) were fully vaccinated patients, demonstrated a 92.0% (95% CI 91.4–92.5%) vaccine efficacy against hospitalisation and a 90.3% (95% CI 88.6–91.8%) vaccine efficacy against mortality [215]. In flu studies, a VE point estimate of 60% for outcomes indicates that the flu vaccine, on average, reduces the risk of these consequences by 60%. In other words, it is more confident that the genuine protective effect of the vaccine is close to 60% if the confidence interval of this point estimate is between 50% and 70% than if it were between 10% and 90% [216].

Real-world VE from all over the world has recently been reported in a number of studies. After the second dosage of the BNT162b2 vaccine, a statewide mass immunisation setting in Israel demonstrated 92% efficacy for verified illnesses [217]. In this regard, the availability of, for instance, COVID-19 vaccines provides policymakers in Low- and Middle-Income Countries (LMICs) with a new tool to fight the pandemic, perhaps reducing the need for lockdowns and other non-pharmaceutical actions. However, there is growing evidence that vaccines are not a magic bullet, so policymakers will need to figure out how to best incorporate vaccines into a larger suite of interventions [218].

Real-world data on the effectiveness of vaccinations in their setting is needed to develop evidence-based policy on the appropriate use of vaccines [219]. Clinical trial efficacy statistics are crucial for regulatory agencies to determine the effectiveness and safety of a vaccination. Nevertheless, there are certain caveats associated with employing efficacy statistics in policymaking. To begin, the groups included in clinical studies are often selected using criteria that are not universally applicable [220]. Second, the local epidemiology may not be reflected in the location of clinical studies. For instance, several clinical studies of the COVID-19 vaccine have been performed in a variety of contexts, each with its own unique circulating strains, underlying population health, transmission dynamics, (non-pharmacological interventions) NPIs, and outcome measures [221]. Monitoring impact, providing country-specific inputs for estimating future vaccination and relaxation of new product introduction (NPI) tactics, and providing justification for money allocation into [222], or away from, the vaccination project (Figure 3) [223]; these are all possible thanks to the results of real-world effectiveness studies. Real-world effectiveness studies require cautious research design because of potential selection bias, confounding factors, and missing data [224].

Concretely, a recent systematic review carried out by Teerawattananon et al. [219] specified that more research is required in LMICs, particularly in Africa and Asia, and that effectiveness studies of longer duration and covering all vaccinations with WHO EUL are required. Additionally, this review pointed out that governments risk losing public support for vaccines if they do not disclose data on vaccine efficacy for all licensed goods. Thirdly, sample sizes were not determined (or reported) beforehand by most investigations [225]. While this may be less of a concern for large-scale retrospective cohort studies using national databases (which can include dozens or millions of records), it is a crucial factor to keep in mind when designing prospective research or conducting smaller-scale retrospective cohort studies. Otherwise, minimum sample size calculations should take into consideration disparities in access to healthcare services and health-seeking behaviours in LMICs, as opposed to high-income countries (HICs), because many LMICs are unlikely to be able to replicate the large-scale studies from HICs [226,227]. Third, there are inconsistencies in how well studies reported missing data and how well they looked for and corrected for potential confounders [228].

The significance of locally collected data on vaccine efficacy offers additional information on key confounders and approaches to manage missing data [229]. Studies on the efficacy of the COVID-19 vaccine have been undertaken mostly in HICs because of the availability of comprehensive and well-connected databases for vaccination, diagnosis, and treatment [230]. However, prospective study designs will be used in LMICs because there are typically no preexisting databases to use, necessitating an up-front determination of sample size and a well-thought-out strategy for dealing with confounders and missing data. In other cases, such as the Burnett et al. review [231], there were inconsistencies from 2006 to 2019 in measurement and reporting; for instance, only 58% of the articles indicated the percentage of enrolled children who had an immunisation card or other documentation of their vaccination status reviewed. In addition, there is a need for such studies for policymakers in LMICs to create and monitor vaccination programmes, and to boost public confidence in vaccination, despite the limited experience conducting vaccine effectiveness in LMICs [232]. Evaluations of the safety, efficacy, and impact of vaccines in the real world in LMICs may be hampered by a lack of comprehensive demographic and disease epidemiology data, as well as a deficient health care and surveillance infrastructure. To determine the best vaccine delivery strategy based on vaccine and country-specific characteristics, a thorough planning phase is necessary [233]. Social mobilisation and a communication strategy are crucial. Implementation research and creative methods used in logistics, delivery, communication, and programme evaluation can reduce the number of new vaccine introductions [234]. Therefore, the international scientific community should help low- and middle-income countries to conduct vaccination effectiveness trials for illnesses in their own countries.

### 13.1. Limitations and Challenges

While biomimetic strategies offer promising avenues to enhance vaccine design, several limitations and challenges must be critically considered to provide a balanced perspective.

First, manufacturing complexity remains a significant hurdle. Biomimetic platforms often rely on advanced nanomaterials, recombinant systems, or modular bioengineered components that demand sophisticated infrastructure and high production costs. Scaling these technologies to meet global vaccine demands, especially in low-resource settings, presents logistical and economic difficulties.

Second, stability and storage pose practical concerns. Some biomimetic constructs—particularly those involving lipid-based nanoparticles or temperature-sensitive adjuvants—exhibit limited shelf-life or require cold-chain logistics that are not always feasible in remote or underserved regions. This may undermine the accessibility and utility of otherwise promising technologies.

Third, immunogenic safety must be carefully evaluated. While mimicking natural immune processes can enhance efficacy, it may also increase the risk of off-target effects, excessive inflammation, or autoimmunity if not properly regulated. Long-term safety profiles of novel biomimetic formulations remain insufficiently characterized, particularly in pediatric, elderly, and immunocompromised populations.

Additionally, regulatory uncertainty is a challenge, as the approval pathways for innovative delivery systems or synthetic bioinspired components are often ambiguous. This may delay the translation of research into clinical practice and complicate global harmonization of vaccine guidelines.

Ethical considerations related to equitable access and distribution of biomimetic vaccines are critical. Special attention must be paid to ensuring availability in low- and middle-income countries (LMICs) and underserved communities, where barriers such as cost, infrastructure, and regulatory disparities often limit vaccine uptake. Moreover, the inclusion of diverse populations in clinical trials is essential to avoid translational gaps and ensure safety and efficacy across different genetic, age, and socioeconomic groups. Addressing these challenges is key to advancing global health equity in the era of precision vaccinology.

Finally, scalability and affordability must be addressed. Technologies that are effective in preclinical models may prove difficult or cost-prohibitive to manufacture at scale, especially in contexts where equitable distribution is a public health priority.

Despite these limitations, ongoing advances in biomanufacturing, regulatory science, and translational research offer potential pathways to overcome these barriers. Acknowledging these challenges is essential to ensure that biomimetic vaccine strategies are not only innovative but also practical, safe, and globally deployable.

### 13.2. Regulatory and Translational Perspectives

From a regulatory and translational standpoint, biomimetic vaccine platforms present unique challenges due to their structural complexity, novel materials, and multicomponent mechanisms of action. Current regulatory frameworks, such as those of the U.S. Food and Drug Administration (FDA) and the European Medicines Agency (EMA), are still evolving in their ability to assess synthetic or bioinspired constructs, especially when they diverge from classical vaccine categories. Key hurdles include the lack of standardized evaluation criteria for novel adjuvants, delivery systems (e.g., nanoparticles, microneedles), and bioengineered antigen platforms. Translating promising preclinical results into human trials is often delayed by uncertainties in toxicology profiling, scalability, and immunogenicity prediction in heterogeneous human populations. To accelerate translation, early engagement with regulatory agencies, harmonization of global standards, and the establishment of dedicated fast-track pathways for advanced vaccine technologies will be essential. These steps will ensure that the innovative potential of biomimetic vaccines is matched by a clear and supportive regulatory trajectory.

## 14. Future Perspectives

As we continue to deepen our understanding of vaccine immunogenicity and effectiveness, several key areas warrant attention for future research and development. First, advancements in vaccine delivery systems, such as novel adjuvants, nanotechnology-based formulations, and alternative routes of administration, hold promise in enhancing immune responses and expanding vaccine coverage [235]. Furthermore, investigating the immunogenicity and effectiveness of vaccines in specific populations, such as immunocompromised individuals, pregnant women, and infants, is crucial for tailoring vaccination strategies to optimize protection in these vulnerable groups [236].

Additionally, exploring the potential of therapeutic vaccines for the treatment of chronic infections and non-communicable diseases represents an exciting avenue for future investigation [237]. Moreover, the development of broadly protective vaccines capable of targeting multiple strains or entire pathogen families could revolutionize disease prevention efforts [238]. Advancements in vaccine monitoring and surveillance systems, including the use of digital health technologies and real-time data analysis, will enable rapid detection of vaccine breakthroughs, emerging variants, and potential waning immunity, facilitating timely intervention and adaptation of vaccination strategies [239].

Lastly, fostering global collaboration and addressing vaccine equity issues are essential for ensuring equitable access to vaccines worldwide and strengthening global health security [240]. One crucial aspect is the development of novel vaccine delivery systems. Ongoing advancements in vaccine technology offer opportunities to enhance immune responses and expand vaccine coverage. The utilization of innovative adjuvants, such as toll-like receptor agonists or nanoparticle-based formulations, holds promise in boosting vaccine efficacy by eliciting stronger and more durable immune responses [235]. Additionally, exploring alternative routes of vaccine administration, such as intranasal or transdermal delivery, could offer advantages in terms of improved mucosal immunity and ease of vaccination [241].

Understanding the immunogenicity and effectiveness of vaccines in specific populations is another important avenue for future research. Vulnerable groups, including immunocompromised individuals, pregnant women, and infants, may have distinct immune responses to vaccines and require tailored vaccination strategies. Investigating the immunological mechanisms underlying vaccine responses in these populations can inform the development of targeted interventions to optimize protection [242,243]. Furthermore, the exploration of therapeutic vaccines for the treatment of chronic infections and non-communicable diseases represents an exciting frontier. Therapeutic vaccines aim to modulate the immune system to control or eliminate persistent infections or to induce immune-mediated responses against tumors. Advancements in understanding the interplay between the immune system and diseases such as HIV, cancer, and autoimmune disorders open possibilities for developing vaccines as therapeutic interventions [244].

Another area of interest is the development of broadly protective vaccines. Current vaccine strategies often target specific strains or variants of pathogens. However, the emergence of new viral strains or pathogen families presents ongoing challenges. The pursuit of vaccines that provide cross-protection against multiple strains or entire pathogen families could revolutionize disease prevention efforts, offering broader and more durable immunity [245]. Advancements in vaccine monitoring and surveillance systems are crucial to ensuring the ongoing effectiveness and safety of vaccination programs. The integration of digital health technologies, real-time data analysis, and sophisticated surveillance methods can facilitate the rapid detection of vaccine breakthroughs, emerging variants, and potential waning immunity. These advancements enable timely intervention, adjustment of vaccination strategies, and targeted public health responses [246].

Lastly, fostering global collaboration and addressing vaccine equity issues are paramount for achieving equitable access to vaccines worldwide and strengthening global health security. Collaborative efforts among countries, organizations, and stakeholders are necessary to ensure fair distribution, affordability, and sustainable vaccine supply chains. Addressing barriers to vaccine access, including socioeconomic disparities and logistical challenges, is vital for achieving universal vaccine coverage and safeguarding public health on a global scale [247,248]. By focusing on these future perspectives, researchers, policymakers, and healthcare professionals can advance our knowledge and overcome existing challenges in the field of vaccine immunogenicity and effectiveness. Such efforts are essential for the development of next-generation vaccines that provide robust and long-lasting protection against infectious diseases, ultimately contributing to improved global health outcomes [249,250,251,252,253].

## 15. Biomimetic Innovations in Vaccine Design: Future Directions

Future research should prioritize the development of microbiota-informed vaccine platforms, exploring how microbial composition and functionality influence vaccine responsiveness across the lifespan. This includes identifying specific bacterial strains or metabolites that enhance immunogenicity and incorporating them into next-generation adjuvants or delivery systems. Additionally, mucosal vaccine delivery—particularly through intranasal or oral routes—presents a promising avenue to induce localized immunity at pathogen entry points. Further investigation into bioinspired mucosal delivery technologies may yield scalable, non-invasive solutions with enhanced protective potential, especially in resource-limited settings. Thus, the future of vaccine development is being reshaped by biomimetic innovations that harness evolutionary principles and natural immune defense strategies. Several emerging directions stand out as particularly promising:

Bioinspired delivery systems: Advanced platforms that mimic viral structures, such as lipid- or protein-coated nanoparticles, are being developed to enhance antigen presentation and promote stronger, longer-lasting immune responses.

Next-generation biomimetic adjuvants: Inspired by pathogen-associated molecular patterns (PAMPs), novel adjuvants such as Toll-like receptor (TLR) agonists aim to activate targeted immune pathways with reduced reactogenicity, improving both efficacy and tolerability.

Personalized and targeted vaccines: The integration of omics technologies and artificial intelligence allows for the identification of individual immune profiles, paving the way for personalized vaccines, particularly beneficial for immunocompromised individuals or those with suboptimal responses to conventional immunization.

Therapeutic vaccines: Beyond prevention, biomimetic strategies are also enabling the development of therapeutic vaccines, aimed at modulating immune responses against chronic infections (e.g., HIV) or non-communicable diseases such as cancer and autoimmune disorders.

Mimicking intercellular communication: Synthetic extracellular vesicles and biohybrid systems are emerging as tools to replicate natural immune signaling pathways, enhancing immunogenicity through biologically intuitive mechanisms.

Alternative administration routes: Intranasal and transdermal vaccine delivery, inspired by natural pathogen entry routes, offer the potential to induce mucosal immunity and support mass immunization efforts through non-invasive methods.

By emulating highly efficient biological processes, biomimetic approaches offer a sustainable and transformative pathway to more effective, safer, and globally accessible vaccines

## 16. Conclusions

The objective of this narrative review was to explore how biomimetic and evolutionary-informed strategies can improve vaccine immunogenicity and effectiveness, particularly by mimicking physiological immune processes and accounting for host-specific factors such as age, genetics, and microbiota composition.

Integrating biomimetic and evolutionary principles into vaccine design represents a promising path toward safer, longer lasting, and more effective immunizations. These strategies are especially relevant for vulnerable populations and global immunization efforts, offering scalable and personalized solutions to current vaccine challenges. By replicating natural immune pathways and addressing individual variability, biomimetic platforms can enhance immune responses while minimizing adverse effects.

However, scientific innovation alone is not sufficient. The successful translation of biomimetic vaccine technologies into public health practice requires coordinated interdisciplinary efforts across basic science, clinical research, regulatory policy, and ethical governance. We call for urgent and sustained investment in translational research to accelerate the development and implementation of these advanced platforms. This includes scalable bioinspired manufacturing methods, inclusive clinical trial designs, and rapid regulatory pathways for novel adjuvants and delivery systems.

Importantly, equitable access must be a foundational pillar in the global deployment of biomimetic vaccines. Efforts should prioritize underserved populations and low- and middle-income countries (LMICs), ensuring that technological advances do not widen existing disparities in immunization coverage. Public–private partnerships, international cooperation, and policy frameworks are essential to guarantee that biomimetic innovations benefit all segments of the global population.

In the face of increasing biological complexity, vaccine hesitancy, and the persistent threat of emerging infectious diseases, it is ethically and scientifically imperative to invest in adaptive, personalized, and inclusive vaccination strategies. Biomimetic approaches offer a pathway to build more resilient and equitable immunization systems—ones that are prepared not only for today’s challenges but for the pandemics of the future.

## Figures and Tables

**Table 1:** Summary of Vaccine Effectiveness.

Study Location	Vaccine(s)	Variant(s)	Effectiveness	Key Finding
United Kingdom	BNT162b2, ChAdOx1	Delta (B.1.617.2)	Reduced effectiveness: BNT162b2 (−13%), ChAdOx1 (−16%). Two doses are comparable to natural infection.	Effectiveness is higher in younger adults; peak viral load is significant for infection risk.
Southern California	Moderna (2 doses)	Delta, Alpha	Delta: 86.7%, Alpha: 98.4%. Declined over time (from 94.1% to 80%).	Effectiveness against Delta wanes over time since vaccination.
Brazil	Ad26.COV2.S (Janssen)	Emerging variants	Symptoms: 50.9%, Hospitalization: 72.9%, ICU: 92.5%, Death: 90.5%	A single dose showed strong protection during variant emergence.
India	2 or 3 doses (type not specified)	Alpha, Beta, Delta, Omicron	The third dose is highly effective at neutralizing variants.	Antibodies declined over 12 months; a booster dose is essential.
Canada	mRNA (single dose)	Alpha, Gamma, other variants	72% (other), 67% (Alpha), 61% (Gamma)	The mRNA vaccine showed only minimal reduction in protection.
South Africa	NVX-CoV2373	B.1.351 (Beta)	Overall: 49.4%, HIV-negative: 60.1%, Variant-specific: 51.0%	Reduced efficacy due to spike protein mutations in the variant.

**Table 2:** Summary of Vaccine Co-Administration Strategies.

Vaccine Combination	Population	Outcome	Key Finding
BCG + DTP	Infants (Bangladesh, India, Senegal)	Reduced all-cause mortality	Lower mortality with co-administration; possible nonspecific immunity via BCG
MMRV + Meningococcal C	Children > 12 months (Europe)	Similar immunogenicity and safety	Co-administration produced a strong immune response to all strains; safe and well-tolerated
Meningococcal C + Seasonal Influenza	Adults (Philippines)	Adequate safety; reduced antibody titers	Safe, but lower meningococcal antibody titers suggest possible immune interference
Meningococcal C + Hepatitis A/B	Adolescents	Optimal safety and efficacy	Strong antibody titers up to 7 months, regardless of co-administration
Meningococcal C + DTaP-HepB-IPV/Hib	Pediatric population	Similar reactogenicity and immunogenicity	No reduction in immunogenicity; acceptable safety profile
COVID-19 + Seasonal Influenza	Adults, the elderly, and at-risk populations	Improved compliance, mixed efficacy signals	Reduced COVID-19 risk in some studies; no major safety concerns; meta-analysis supports modest protection

## Data Availability

Not applicable.
